# Advancing entity recognition in biomedicine via instruction tuning of large language models

**DOI:** 10.1093/bioinformatics/btae163

**Published:** 2024-03-21

**Authors:** Vipina K Keloth, Yan Hu, Qianqian Xie, Xueqing Peng, Yan Wang, Andrew Zheng, Melih Selek, Kalpana Raja, Chih Hsuan Wei, Qiao Jin, Zhiyong Lu, Qingyu Chen, Hua Xu

**Affiliations:** Section of Biomedical Informatics and Data Science, School of Medicine, Yale University, New Haven, CT-06510, United States; McWilliams School of Biomedical Informatics, University of Texas Health Science at Houston, Houston, TX-77030, United States; Section of Biomedical Informatics and Data Science, School of Medicine, Yale University, New Haven, CT-06510, United States; Section of Biomedical Informatics and Data Science, School of Medicine, Yale University, New Haven, CT-06510, United States; Section of Biomedical Informatics and Data Science, School of Medicine, Yale University, New Haven, CT-06510, United States; William P. Clements High School, Sugar Land, TX-77479, United States; Stephen F. Austin High School, Sugar Land, TX-77498, United States; Section of Biomedical Informatics and Data Science, School of Medicine, Yale University, New Haven, CT-06510, United States; National Center for Biotechnology Information, National Library of Medicine, National Institutes of Health, Bethesda, MD-20894, United States; National Center for Biotechnology Information, National Library of Medicine, National Institutes of Health, Bethesda, MD-20894, United States; National Center for Biotechnology Information, National Library of Medicine, National Institutes of Health, Bethesda, MD-20894, United States; Section of Biomedical Informatics and Data Science, School of Medicine, Yale University, New Haven, CT-06510, United States; National Center for Biotechnology Information, National Library of Medicine, National Institutes of Health, Bethesda, MD-20894, United States; Section of Biomedical Informatics and Data Science, School of Medicine, Yale University, New Haven, CT-06510, United States

## Abstract

**Motivation:**

Large Language Models (LLMs) have the potential to revolutionize the field of Natural Language Processing, excelling not only in text generation and reasoning tasks but also in their ability for zero/few-shot learning, swiftly adapting to new tasks with minimal fine-tuning. LLMs have also demonstrated great promise in biomedical and healthcare applications. However, when it comes to Named Entity Recognition (NER), particularly within the biomedical domain, LLMs fall short of the effectiveness exhibited by fine-tuned domain-specific models. One key reason is that NER is typically conceptualized as a sequence labeling task, whereas LLMs are optimized for text generation and reasoning tasks.

**Results:**

We developed an instruction-based learning paradigm that transforms biomedical NER from a sequence labeling task into a generation task. This paradigm is end-to-end and streamlines the training and evaluation process by automatically repurposing pre-existing biomedical NER datasets. We further developed BioNER-LLaMA using the proposed paradigm with LLaMA-7B as the foundational LLM. We conducted extensive testing on BioNER-LLaMA across three widely recognized biomedical NER datasets, consisting of entities related to diseases, chemicals, and genes. The results revealed that BioNER-LLaMA consistently achieved higher F1-scores ranging from 5% to 30% compared to the few-shot learning capabilities of GPT-4 on datasets with different biomedical entities. We show that a general-domain LLM can match the performance of rigorously fine-tuned PubMedBERT models and PMC-LLaMA, biomedical-specific language model. Our findings underscore the potential of our proposed paradigm in developing general-domain LLMs that can rival SOTA performances in multi-task, multi-domain scenarios in biomedical and health applications.

**Availability and implementation:**

Datasets and other resources are available at https://github.com/BIDS-Xu-Lab/BioNER-LLaMA.

## 1 Introduction

The current paradigm in Biomedical Natural Language Processing (BioNLP) predominantly relies on the utilization of pre-trained domain-specific language models such as Bidirectional Encoder Representations from Transformers (BERT) (Devlin *et al.* 2018). These models are trained on large-scale biomedical texts, such as PubMed articles and clinical notes, to develop a comprehensive understanding of the specialized language used in the biomedical field (Lee *et al.* 2020, Gu *et al.* 2021). Subsequently, these models are fine-tuned for specific tasks, such as named entity recognition (NER), to extract entities like genes, diseases, and chemicals from biomedical texts. This fine-tuning process typically focuses on a single task, although some efforts have explored multi-task learning (MTL) (Peng *et al.* 2020, Yadav *et al.* 2022) and an all-in-one scheme (Luo *et al.* 2023), where a single model is trained to perform multiple related tasks. MTL leverages shared representations and shared knowledge across tasks by learning to jointly optimize the shared parameters to minimize the total loss across all tasks thereby improving the overall performance (Crichton *et al.* 2017, Wang *et al.* 2019). The all-in-one strategy consolidates multiple datasets with different entity types into a unified format and utilizes this dataset to train a model (Luo *et al.* 2023).

Nevertheless, despite the considerable progress made with these approaches, there are still some notable limitations. For instance, the performance of domain-specific fine-tuned BERT models may degrade when applied to out-of-domain tasks because it may not capture the specific context and vocabulary of the new domain as effectively (Košprdić *et al.* 2023). If the training data used to create the model does not capture the variations, specificities, or niche aspects of the domain, the model’s performance may be limited when dealing with sub-domains that differ significantly from the ones it was trained on (Khambete *et al.* 2021). If the fine-tuning approach is task-specific, the learned knowledge cannot always be easily transferred to new tasks. These models also present some difficulties in using existing smaller datasets for different tasks and combining them to create larger datasets for multi-task or cross-domain training. For example, in the case of NER, datasets are curated in different standards (e.g. different entity types, spans of tokens, and normalizations). Simply combining the datasets results in inconsistent annotations with the same entity annotated in one dataset and omitted in the other, affecting the quality of the dataset and in turn the model performance. These limitations have served as a motivation for exploring innovative approaches that have the potential to enhance the current BioNLP learning paradigm.

Recently, generative pre-trained transformer (GPT) models with billion scale parameters such as GPT-3.5 (https://platform.openai.com/docs/models/gpt-3-5) and GPT-4 (Achiam *et al.* 2023) have achieved state-of-the-art results on a wide range of NLP tasks, including text classification, summarization, question answering, and translation especially in zero or few-shot setting, which may potentially address the limitations mentioned above (Gilardi *et al.* 2023, Gilson *et al.* 2023, Hendy *et al.* 2023, Loukas *et al.*, 2023, Manakhimova *et al.* 2023, Wang *et al.* 2023a, 2023b). These large language models (LLMs) require huge computational resources to train and are often trained on massive amounts of data including proprietary datasets. LLMs have also been shown to be able to learn from human feedback, which allows them to improve their performance over time. In recent months, there has also been a rise in the availability of more open source LLMs, such as LLaMA 1 and 2 (Touvron *et al.* 2023a, 2023b), Falcon LLM (https://falconllm.tii.ae/), etc. Although these models generally do not match the proficiency of the most advanced closed models, their capabilities have been quickly improving. It has also been found that the use of well curated training data can improve the performance of these models. This has eventually led to the development of instruction-following models such as Stanford Alpaca (https://crfm.stanford.edu/2023/03/13/alpaca.html), Koala (https://bair.berkeley.edu/blog/2023/04/03/koala/), and Vicuna (https://lmsys.org/blog/2023-03-30-vicuna/), which instruction fine-tuned the LLaMA base model on data generated using GPT models (text-davinci-003), dialogue data gathered from the web, and user-shared conversations collected from ShareGPT, respectively.

Previously, in a pilot study (Chen *et al.* 2023) we evaluated the performance of GPT-3.5 and GPT-4 on 12 BioNLP datasets across 6 tasks namely NER, relation extraction, multi-label document classification, question answering, text summarization, and text simplification. Our results showed that compared to other tasks, the performance of GPT models was considerably lower on NER datasets (e.g. NCBI-disease Doğan *et al.* 2014) when compared to a fine-tuned PubMedBERT (Gu *et al.* 2021) model. Other studies have also observed consistent results (Agrawal *et al.* 2022, Hu *et al.* 2024). LLMs are predisposed towards generative tasks such as question answering and text summarization that do not require precise localization and categorization of entities found within documents. As a result, these models have not measured up as effectively on NER as other supervised approaches designed specifically for the nuances of the sequence labeling framework inherent to this critical information extraction task. Hence, in this study, we developed an instruction finetuned model (BioNER-LLaMA) and compare its performance with fine-tuned PubMedBERT models and an existing medical-specific LLM (PMC-LLaMA) (Wu *et al.* 2023). In the instruction tuning framework, it is possible to combine multiple available datasets and only make modifications to the prompts to extract a specific entity type or multiple entities. We leveraged three existing annotated NER corpora with three different entity types, transforming them into instruction demonstrations and combining them to train an instruction-following model capable of achieving strong performance across different NER datasets.

We provide several contributions in the current work. First, we introduce an approach for developing instruction-following models applicable to biomedical NER by deriving instruction demonstrations directly from existing annotated biomedical NER corpora rather than utilizing instruction samples generated by ChatGPT or humans. Second, we demonstrate that a single NER model refined via our proposed instruction tuning paradigm achieves performance comparable to entity-type-specific PubMedBERT model separately fine-tuned on three biomedical entity categories. Where previous methods required distinct, specialized models per entity type, our unified instruction-guided technique accomplishes this task with a single trained system. Next, we compare the performance of PMC-LLaMA, a LLaMA based LLM that has been specifically designed for medical applications to BioNER-LLaMA. Finally, we analyze the impact of instruction dataset size and prompt structure on the capabilities of our instruction-following NER model. Through systematically varying these parameters, we provide valuable insight into optimizing the proposed methodology and realizable degrees of performance depending on training resources. In addition, we make publicly available all the instruction datasets created as a resource to the community. Overall, our work presents important initial strides toward a generalizable framework for biomedical NER through instruction tuning of LLMs with a potential of applying the same to other biomedical NLP tasks.

## 2 Background

### 2.1 Biomedical named entity recognition

The methodology for NER has progressed significantly over the years from rule-based to feature-driven statistical learning to fully end-to-end neural networks, greatly reducing manual effort through self-supervised pretraining. Early systems relied heavily on rule-based approaches using dictionaries and regular expressions to match entity mentions with one of the main constraints being the need for extensive domain knowledge engineering (Fukuda *et al.* 1998, Tsuruoka and Tsujii 2003, Yang *et al.* 2008). Statistical machine learning approaches like Hidden Markov Model (Morwal *et al.* 2012) and Conditional Random Field (CRF) (Sutton and McCallum 2012) were then introduced, which along with various embedding techniques reduced dependency on hand-crafted features and rules to some extent (Tsai *et al.* 2006, Ponomareva *et al.* 2007, Shao *et al.* 2021). Systems such as ABNER (A Biomedical Named Entity Recognizer) (Settles 2005) utilized CRF to achieve improved performance over the rule-based systems. With the rise of deep neural networks, approaches such as BiLSTM-CRF (Huang *et al.* 2015) which combines LSTM networks with CRF layers, removed reliance on feature engineering (Dang *et al.* 2018, Cho and Lee 2019). Later, pretrained language models, such as BERT (Devlin *et al.* 2018), based on the Transformer architecture significantly advanced the field by learning rich linguistic representations from large unlabeled text. Domain-specific pretraining using massive biomedical corpora, as in BioBERT (Lee *et al.* 2020) and SciBERT (Beltagy *et al.* 2019), followed by fine-tuning on downstream tasks has helped push the performance of these models achieving state-of-the-art results on various tasks and datasets. Current research also utilizes massive pretraining and MTL to leverage diverse biomedical sources and tasks simultaneously (Weber *et al.* 2020, Zhou *et al.* 2021, Chaudhry *et al.* 2022, Rodriguez *et al.* 2022). MTL enables the joint training of multiple tasks, promoting knowledge sharing and improving performance across related tasks. Crichton *et al.* (2017) trained and evaluated a multi-task CNN on 15 domain-specific datasets representing several biomedical named entities. More recently, the all-in-one NER (Luo *et al.* 2023) approach integrated heterogeneous biomedical entity datasets into a single format to train a model that can recognize multiple entity types.

### 2.2 Large language models

Research in recent years has shown improved performance on varied tasks by scaling pretrained language models. With the increase in the number of parameters these large models with tens of billions of parameters, apart from achieving significant improvements in performance are also shown to exhibit better zero/few-shot learning capabilities on unseen tasks (Kojima *et al.* 2022, Zhao *et al.* 2022). To distinguish between language models of varying sizes, the research community uses the term “large language models” or LLMs to refer to pretrained models that have a significantly large number of parameters, such as those containing tens or hundreds of billions of parameters. The release of GPT-3.5 and GPT-4 has transformed and reoriented the way we develop AI algorithms. With the restricted access of these models limiting the ability of the research community we have also seen a push towards smaller open-source LLMs like Meta’s LLaMA (https://ai.meta.com/blog/large-language-model-llama-meta-ai/) and EleutherAI’s Pythia (Biderman *et al.* 2023).

### 2.3 NER using LLMs

For domains which are highly diverse such as biomedicine, only a few high-quality datasets are available due to high annotation costs and scarce domain expertise. Hence zero-shot and few-shot in-context learning capabilities of LLMs that require no training data or a few examples in the form of instruction demonstrations are highly appealing. Recent research has evaluated the performance of GPT-3, 3.5, and GPT-4 models on NER datasets and compared the performance with supervised BERT models and its domain-specific variants (Ashok and Lipton 2023, Wang *et al.* 2023c, Wei *et al.* 2023, Hu *et al.* 2024). While these models do not generally achieve similar performance as supervised models, it has been shown that customizing and enhancing the prompts based on the tasks, for example with entity definitions, specific annotation guidelines, etc. achieve much better performance compared to simple prompts (Hu *et al.* 2024). With the introduction of instruction-following models like Stanford Alpaca and Vicuna, various frameworks for NER have been proposed using these models (Ji 2023, Labrak *et al.* 2023). For example, VicunaNER (Ji 2023) is a two-phase framework for conducting both zero/few-shot NER using Vicuna. In the “recognition” phase multi-turn dialogues with Vicuna first identifies the entities and then checks for its correctness. The second phase, “re-recognition” further performs a multi-turn dialogue to recognize entities unrecognized in the first phase. Zhou *et al.* (2023) used ChatGPT to generate instruction tuning data from unlabeled web text and conducted instruction tuning on LLaMA.

## 3 Materials and methods

### 3.1 Instruction tuning

**Figure 1.**
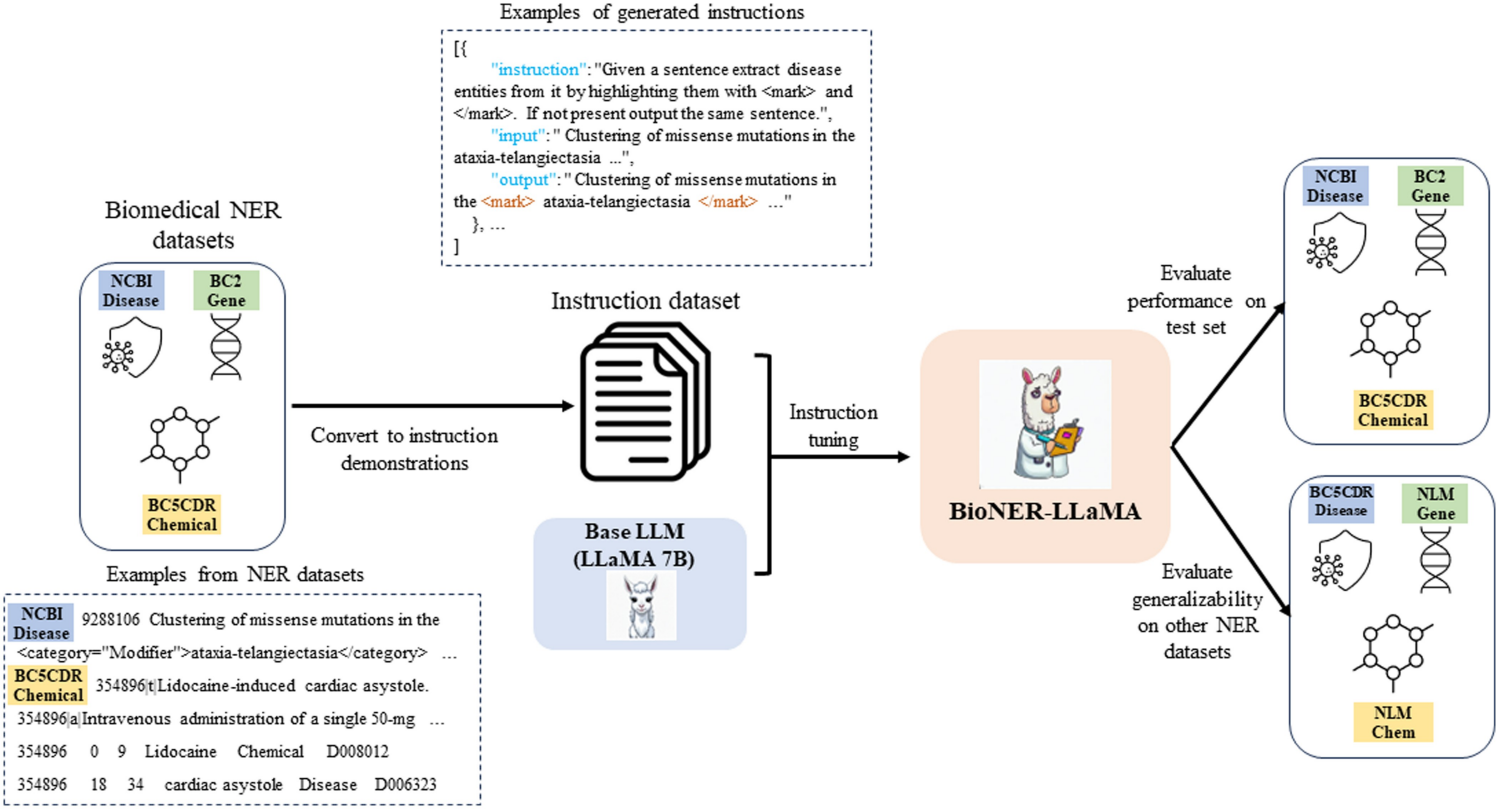
Framework for the development of instruction tuned large language models utilizing existing NER datasets.

Instruction tuning, also referred to as instruction finetuning is a process that involves supervised training of language models to adhere to instructions in order to accomplish a specific task. First introduced by Wei *et al.* (2021), this approach was shown to substantially improve zero-shot performance on unseen tasks. This has been further substantiated by models such as Stanford Alpaca (https://crfm.stanford.edu/2023/03/13/alpaca.html), Flan-UL2 (https://huggingface.co/google/flan-ul2), etc. We used the LLaMA 7B model (LLaMA 1 Touvron *et al.* 2023a and LLaMA 2 Touvron *et al.* 2023b) and instruction tuned it using publicly available biomedical NER datasets by converting these datasets to natural language instruction templates. The NER datasets were selected to include three different biomedical entity types—disease, chemical, and gene. The workflow for the development of the instruction tuned model (BioNER-LLaMA) is illustrated in Fig. 1. We describe the datasets used for instruction tuning, the instruction tuning procedure and other NER datasets used for evaluating the generalizability of the model in detail below.

#### 3.1.1 Datasets for instruction tuning

For this study, we focused on a single task, i.e. NER with datasets covering three different entity types. A detailed description of the datasets is provided below.

**NCBI disease:** The NCBI disease corpus (Doğan *et al.* 2014) is a collection of 793 PubMed abstracts manually annotated with disease mentions and their corresponding MeSH or OMIM concept identifiers. The corpus is split into training, development, and test sets with a total of 6892 disease mentions mapped to 790 unique identifiers.

**BC5CDR-Chemical:** The BioCreative V Chemical-Disease Relations corpus (Li *et al.* 2016) consists of a total of 1500 PubMed abstracts split into subsets of 500 each for training, development, and test. The corpus is annotated for disease mentions, chemical mentions, and chemical-disease interactions. For instruction tuning we only retained annotated chemical mentions for creating instruction examples. We used the disease annotations as part of other datasets for evaluating generalizability (see below).

**BC2GM:** The BioCreative II Gene Mention (Smith *et al.* 2008) corpus is concerned with the extraction of gene and gene product mentions in MEDLINE sentences. There are a total of 20 000 sentences divided into train (12 500 sentences), development (2500), and test splits (5000).

#### 3.1.2 Instruction tuning procedure

The two important requirements for developing an instruction tuned model are a strong pretrained language model and a good quality dataset with instruction-following examples. For the pretrained language model we utilized Meta’s LLaMA-1 7B and LLaMA-2 7B models. The above-mentioned three NER datasets were used to create instruction examples. The abstracts were split into sentences and each sentence was used as an “input” to the model. The spaCy “sentencizer” was used for sentence boundary detection and segmentation. The dataset was created as a JSON file with a list of dictionaries, with each dictionary containing three fields: instruction, input, and output. The “instruction” describes a task the model should perform. The “input” is a sentence from the training set of any of the three datasets and can possibly have zero, one or more entity mentions. The “output” is the sentence demarcating the entities with <mark> and </mark> tags if entities are present, or the exact input sentence otherwise. Formatting the output of the model this particular way, enables easy postprocessing and conversion to common tagging formats (e.g. BIO format) used for NER. This also brings in the added advantage of utilizing existing evaluation scripts for NER tasks.

**Figure 2.**
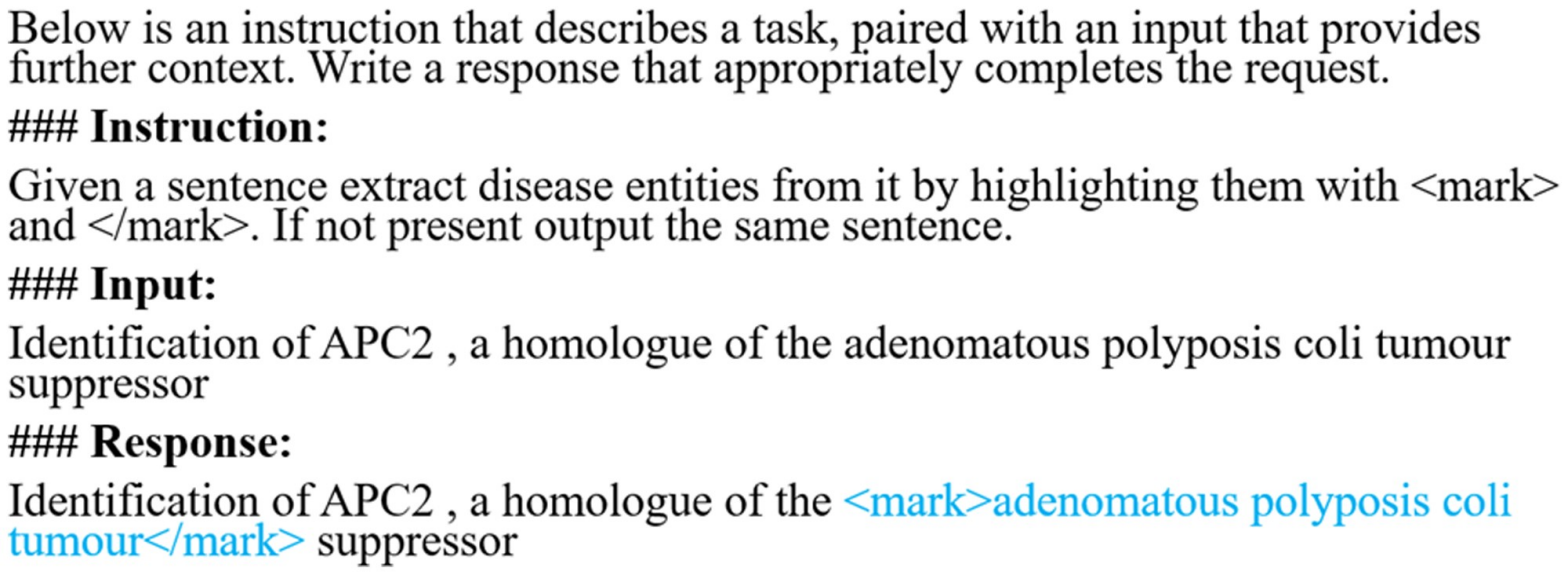
A prompt example used for instruction tuning to extract a disease mention (constructed from NCBI disease dataset).

An example prompt used to extract disease mentions utilizing the NCBI disease training set is shown in Fig. 2. A similar prompt replacing the word “disease” in the “instruction” with “chemical,” and “gene” was used when transforming the training set of BC5CDR-Chemical, and BC2GM, respectively into instruction examples. Thus, our instruction dataset used for fine-tuning consists of instruction examples generated by transforming the training set of all three datasets.

With the pretrained language models and datasets in hand, we proceeded to fine tune the models to obtain BioNER-LLaMA1 and BioNER-LLaMA2 (corresponding to LLaMA-1 and LLaMA-2 models). Our fine-tuning followed the Stanford Alpaca approach using Hugging Face’s training framework utilizing fully sharded data parallel and mixed precision training. During inference, we provided the fine-tuned models with “instruction” and “input” to generate the “output.”

### 3.2 Baselines

**PubMedBERT:** A pretrained biomedical BERT model, PubMedBERT (Gu *et al.* 2021) was selected as a baseline. PubMedBERT is pretrained from scratch using abstracts and full-text articles from PubMed and PubMed Central (PMC). This model has achieved top performance on several biomedical NLP tasks and benchmarks (Gu *et al.* 2021, Li *et al.* 2022, Fang *et al.* 2023). For this study we fine-tuned PubMedBERT using the training and validation splits of the three NER datasets. Hyperparameter tuning was performed using different values for learning rate (1e-05, 3e-5, 5e-5), sequence length (128, 256, 512), batch size (16, 32) and dropout rate (0.1) to select the model that achieved the best loss on validation set. This is consistent with the original PubMedBERT study (Gu *et al.* 2021). Each dataset was fine-tuned using these 18 combinations of hyperparameters and minimum, median, and maximum performance was calculated.

**GPT Zero-shot/Few-shot:** We evaluated the performance of GPT 3.5 (GPT-3.5-turbo-0301) and GPT 4 (GPT-4–0314) on the test sets of all three NER datasets used for instruction tuning. Both zero-shot and five-shot learning performance was evaluated for both the models. For five-shot learning we randomly selected five examples from the training set of the respective dataset. The same prompt that was used for instruction tuning (Fig. 2) was also used for these experiments.

**PMC-LLaMA:** PMC-LLaMA (Wu *et al.* 2023) is an open-source language model designed specifically for medical applications. The training of PMC-LLaMA consisted of two parts: (i) a data-centric knowledge injection step by integrating 4.8M biomedical academic papers and 30K medical textbooks and (ii) an instruction tuning step with instruction samples focusing on medical conversation, medical rationale question-answering, and QA pairs from translating knowledge in knowledge graphs. PMC-LLaMA consists of 13B parameters. Even though PMC-LLaMA is an instruction following model, it was not instruction tuned specifically on NER samples as can be inferred from (ii) above. Initial experiments using this model demonstrated poor results and hence we instruction tuned this model on our instruction dataset before evaluating the performance.

### 3.3 Evaluating generalizability

To evaluate how our fine-tuned model would generalize to other datasets, we used another set of three publicly available NER datasets, the details of which are described below. The test splits of these datasets converted to instruction examples were used to generate responses from the fine-tuned model and used for performance evaluation.

**BC5CDR-Disease:** As mentioned above, the BioCreative V Chemical-Disease Relations corpus (Li *et al.* 2016) is annotated for disease mentions, chemical mentions, and chemical-disease interactions. We utilized the chemical annotations for fine-tuning the model. A separate dataset with only disease mention annotations was used for evaluation.

**NLM-Chem-BC7:** The NLM Chem BioCreative VII (NLM-Chem-BC7) corpus (Islamaj *et al.* 2022) consists of full-text PMC articles annotated for chemical entities and mapped to MeSH.

**NLM-Gene:** The NLM-Gene corpus (Islamaj *et al.* 2021) is an annotated corpus for genes covering gene mentions of different species (e.g. humans, rats, etc.). This corpus consists of 550 PubMed abstracts split into 450 abstracts for training and 100 abstracts for testing.

### 3.4 Evaluation metrics

Precision, recall, and F1 scores were calculated for each entity type. We calculated the exact match (strict) precision, recall, and F1 score for all the models. In the case of zero/few-shot GPT models, calculating strict F1 score may underestimate the performance of the model as it penalizes even the slightest misalignments of entity boundaries which may not necessarily indicate an incorrect entity. We discuss this in detail in the Results section and provide a comparison based on partial match F1 score.

### 3.5 Additional analysis

We performed several experiments to further evaluate the performance of the model by (i) varying the number of instruction instances in the dataset and (ii) creating separate models for each dataset, and (iii) providing additional information about the entity as part of the “### Instruction” rather than the generic prompt (as in Fig. 2).

To determine the effect of the size of instruction tuning dataset on the performance of the model, we experimented by considering subsets of the training split from all three datasets. For each experiment we randomly selected X% (e.g. 50%, 25%, 10%) from each of the datasets and combined them to create an instruction dataset having all three entity types.

Prior work (Wei *et al.* 2021) has demonstrated that instruction tuning performance improves with the addition of multiple datasets and tasks. While our task is limited to NER, we investigated how the performance varies when instruction tuning on a single dataset (with a single entity type) versus multiple datasets (multiple entity types). Three separate models were developed, each fine-tuned via instructions solely from one dataset, comparing them to BioNER-LLaMA which obtained instructions from three combined datasets.

Additionally, another set of experiments was executed in which the “instruction” part of the prompt was revised to include more information from the annotation guidelines about the entity to be extracted (provided as part of the original dataset). For example, for the instruction demonstrations constructed from the NCBI disease dataset, the following sentence was appended to the “### Instruction”: “Extract all mentions referring to a disease including specific diseases, disease classes or composite mentions of diseases.” A prior study (Hu *et al.* 2024) has shown that prompts with additional information about entities performed better when compared to a basic prompt (which only stated what entities to extract) using GPT 3.5. While this could be the case with a zero-shot/few-shot setting, we wanted to explore whether this translates into the current scenario where we are instruction tuning LLMs.

## 4 Results

The instruction dataset we used for fine-tuning consists of 22 484 instruction-following demonstrations constructed from the training split of NCBI disease (5424 instruction instances, 5132 mentions, 2686 instances with no entity mentions), BC5CDR-Chemical (4560 instruction instances, 5203 mentions, 1609 instances with no entity mentions), and BC2GM (12 500 instruction instances, 15 195 mentions, 6113 instances with no entity mentions) datasets. The test split consists of 940 instances with 957 mentions for NCBI disease, 4797 instances with 5385 mentions for BC5CDR-Chemical, and 5000 instances with 6324 mentions for BC2GM.

### 4.1 Model performance

The performance of all the models along with our BioNER-LLaMA models on the test splits of the three NER datasets is shown in Table 1. The performance of GPT models is lower compared to PubMedBERT and LLaMA-based models. We note that the same prompt (see Fig. 2) was used to evaluate all LLMs. As the performance of GPT models are heavily dependent on the prompts, we explore in the Discussion section several reasons for the low performance and possible solutions that may improve the performance. BioNER-LLaMA models achieved comparable performance to that of fine-tuned PubMedBERT models. BioNER-LLaMA achieved slightly better performance on two datasets (even though the results are not statistically significant), while PubMedBERT model performed better on chemical entity extraction (BC5CDR Chemical dataset). PMC-LLaMA achieved lower F1 scores compared to both PubMedBERT and BioNER-LLaMA models. It should be noted that PubMedBERT and PMC-LLaMA are pretrained on PubMed articles. Furthermore, we are comparing the performance of a single instruction tuned model with three separate fine-tuned PubMedBERT models each fine-tuned on one entity type. Among the BioNER-LLaMA models, the performance is similar with less than 0.5% variability in most cases.

**Table 1.** Precision, recall, and F1 scores for all models.

Model	Dataset			
	Strict/partial match	NCBI (disease)	BC5CDR (chemical)	BC2GM (gene)
		P/R/F1	P/R/F1	P/R/F1
GPT-3.5 (zero-shot)	Strict	0.483/0.286/0.359	0.743/0.448/0.559	0.302/0.430/0.355
	Partial	0.707/0.425/0.531	0.860/0.521/0.649	0.507/0.715/0.593
GPT-3.5 (five-shot)	Strict	0.515/0.230/0.318	0.235/0.721/0.355	0.450/0.351/0.395
	Partial	0.571/0.260/0.358	0.256/0.781/0.385	0.725/0.517/0.603
GPT-4 (zero-shot)	Strict	0.484/0.502/0.493	0.814/0.668/0.734	0.406/0.473/0.437
	Partial	0.665/0.723/0.692	0.899/0.749/0.818	0.689/0.752/0.719
GPT-4 (five-shot)	Strict	0.515/0.682/0.586	0.788/0.835/0.811	0.459/0.536/0.494
	Partial	0.589/0.789/0.675	0.837/0.882/0.859	0.755/0.791/0.772
PubMedBERT	Strict	0.868/0.877/0.873	0.924/0.942/**0.933**	0.845/0.816/0.830
	Partial	0.915/0.936/0.926	0.945/0.967/**0.956**	0.967/0.931/0.949
PMC-LLaMA	Strict	0.877/0.851/0.864	0.935/0.870/0.902	0.834/0.823/0.828
	Partial	0.938/0.914/0.926	0.957/0.892/0.924	0.959/0.943/0.951
BioNER-LLaMA1	Strict	0.869/0.882/0.876	0.935/0.916/0.926	0.841/0.837/**0.839**
	Partial	0.935/0.954/0.944	0.956/0.938/0.947	0.958/0.952/0.955
BioNER-LLaMA2	Strict	0.874/0.886/**0.880**	0.936/0.920/0.928	0.832/0.836/0.834
	Partial	0.941/0.957/**0.949**	0.957/0.944/0.951	0.955/0.956/**0.956**

Numbers in bold represents the best performance for each dataset.

The strict F1 metric penalizes minor deviations in entity boundaries, whereas partial F1 score also considers partial matches between predicted and gold entity boundaries. On an average, for BioNER-LLaMA2 model when evaluated using partial F1 score, there was a 7.13% increase across all the three NER datasets with BC2GM dataset recording a maximum difference of 12.2% [0.834 (strict) versus 0.956 (partial)]. This difference was also reflected in the PubMedBERT model performance [0.830 (strict) versus 0.949 (partial); 11.9% increase] and 5-shot GPT-4 model performance [0.494 (strict) versus 0.772 (partial) 27.8% increase] on BC2GM dataset. This high difference could potentially also signal towards some inconsistencies in the annotations in this dataset. The smallest difference was observed for BC5CDR-Chemical dataset with a 2.3% increase for BioNER-LLaMA model, 2.3% increase for fine-tuned PubMedBERT model, and 4.8% increase using 5-shot GPT-4.

**Figure 3.**
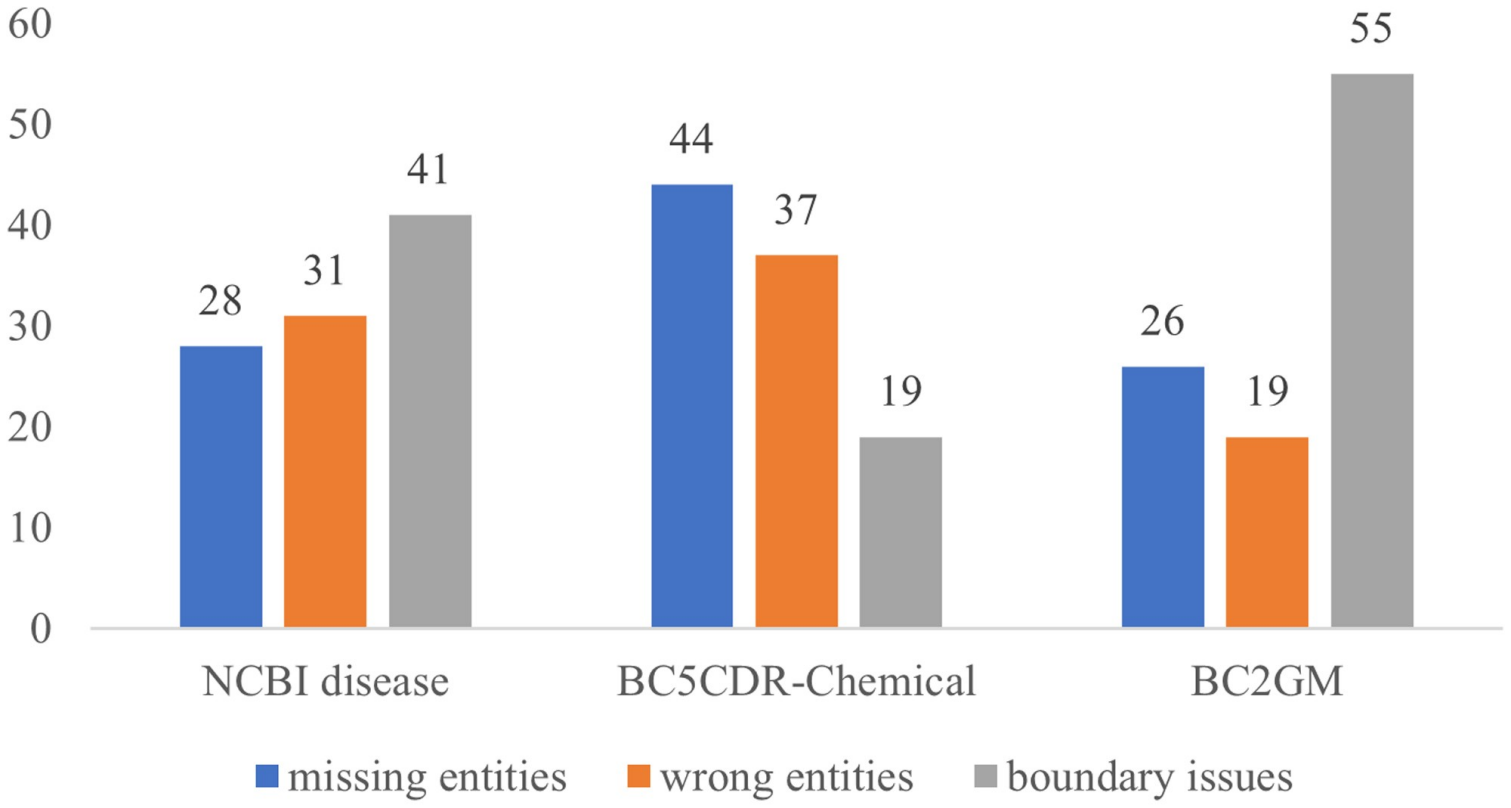
Error analysis on 100 samples of BioNER-LLaMA predictions

We performed a manual error analysis (Fig. 3) on a random sample of 100 instances and categorized the errors in BioNER-LLaMA predictions into three types based on (Chen *et al.* 2023): (i) missing entities, where gold-standard entities are missed by the model, (ii) wrong entities, where incorrect entities are predicted by the model, and (iii) boundary issues: where the predicted entities are correct but with different text spans than the gold standard. Figure 3 shows the distributions. While most of the errors resulted from partial overlap between manually annotated and predicted entities, there were several cases where the models missed some entities (major category for BC5CDR-Chemical) or predicted some entities that were not annotated. Abbreviations constituted a major part of false positive and false negative predictions. For example, the disease spinocerebellar ataxias type 1, 2, and 3 (abbreviated as SCA1, SCA2, and SCA3) was always predicted as “O” (denoting outside of the entity) by the model. Conversely, PDS (disease gene), hp2, and hpdel (allelic genes) were predicted as diseases. BioNER-LLaMA also predicted any phrases that contained deficiency/insufficiency (e.g. smad-4 deficient, haploinsufficiency) as a disease while the manual annotations did not consider these as disease.

Analysis of a random sample of 100 instances of errors in PubMedBERT predictions demonstrated the same type of errors as mentioned above. For example, for the NCBI disease dataset predictions, there were 19 missing entities, 37 wrong entities, and 44 boundary issues. Similar to BioNER-LLaMA predictions, abbreviations, and terms with deficiency (e.g. MLH1 deficiency) resulted in a lot of missing predictions and wrong predictions, respectively apart from the boundary issues. There were some cases where BioNER-LLaMA predictions were same as the gold standard while PubMedBERT predictions were not. For example, cases that mentioned multiple syndromes together were annotated as a single entity (e.g. *Saethre—Chotzen*, *Crouzon*, *and Pfeiffer syndromes* was tagged as BIIIIIII) in the NCBI dataset. PubMedBERT model predicted them as three different entities (tagged as BIIOBOOBI).

### 4.2 Evaluation of performance on other NER datasets

Table 2 presents the outcomes obtained by applying BioNER-LLaMA, PMC-LLaMA, and PubMedBERT models to the other three datasets not used for finetuning. The results for PubMedBERT are from three models, one for each entity type. For example, the model fine-tuned on NCBI disease dataset was evaluated on the test set of BC5CDR-Disease. A similar approach matching the entity types was performed for the other two datasets. Conversely, only the instruction was changed to extract the corresponding entity for the BioNER-LLaMA and PMC-LLaMA models. The test set of BC5CDR-Disease consisted of 4797 instances with 4424 disease mentions. For the NLM-Chem test split there were 10 916 instances with 11 799 chemical mentions and for NLM-Gene there were 1128 instances with 2736 gene mentions.

**Table 2.** Performance of all models on the other three NER datasets.

Model	Dataset			
	Strict/partial match	BC5CDR (disease)	NLM (chemical)	NLM (gene)
		P/R/F1	P/R/F1	P/R/F1
PubMedBERT	Strict	0.748/0.557/0.639	0.862/0.652/**0.742**	0.830/0.795/0.812
	Partial	0.896/0.677/0.771	0.901/0.680/**0.775**	0.926/0.881/0.902
PMC-LLaMA	Strict	0.750/0.529/0.620	0.887/0.571/0.695	0.890/0.756/0.818
	Partial	0.897/0.625/0.737	0.923/0.589/0.719	0.972/0.825/0.892
BioNER-LLaMA1	Strict	0.761/0.577/0.657	0.869/0.589/0.702	0.882/0.765/0.819
	Partial	0.925/0.693/0.793	0.897/0.606/0.723	0.974/0.841/0.902
BioNER-LLaMA2	Strict	0.740/0.610/**0.669**	0.853/0.598/0.703	0.881/0.770/**0.822**
	Partial	0.903/0.736/**0.811**	0.882/0.617/0.726	0.969/0.846/**0.903**

Numbers in bold represents the best performance for each dataset when tested for generalizability.

The BioNER-LLaMA2 model performed better than the fine-tuned PubMedBERT and PMC-LLaMA models on the BC5CDR disease dataset. There is a difference of more than 10% between the strict and partial match F1 scores for this dataset for all the models. This difference can be explained by analyzing the annotation guidelines for these datasets (see Discussion). For the chemical entity extraction on NLM Chem dataset, PubMedBERT performed better than other models. Among the three datasets tested for generalizability, NLM-Gene dataset had the best strict match F1 score with BioNER-LLaMA2 achieving 0.822.

### 4.3 Additional analysis

#### 4.3.1 Effect of size of instruction dataset on performance

**Figure 4.**
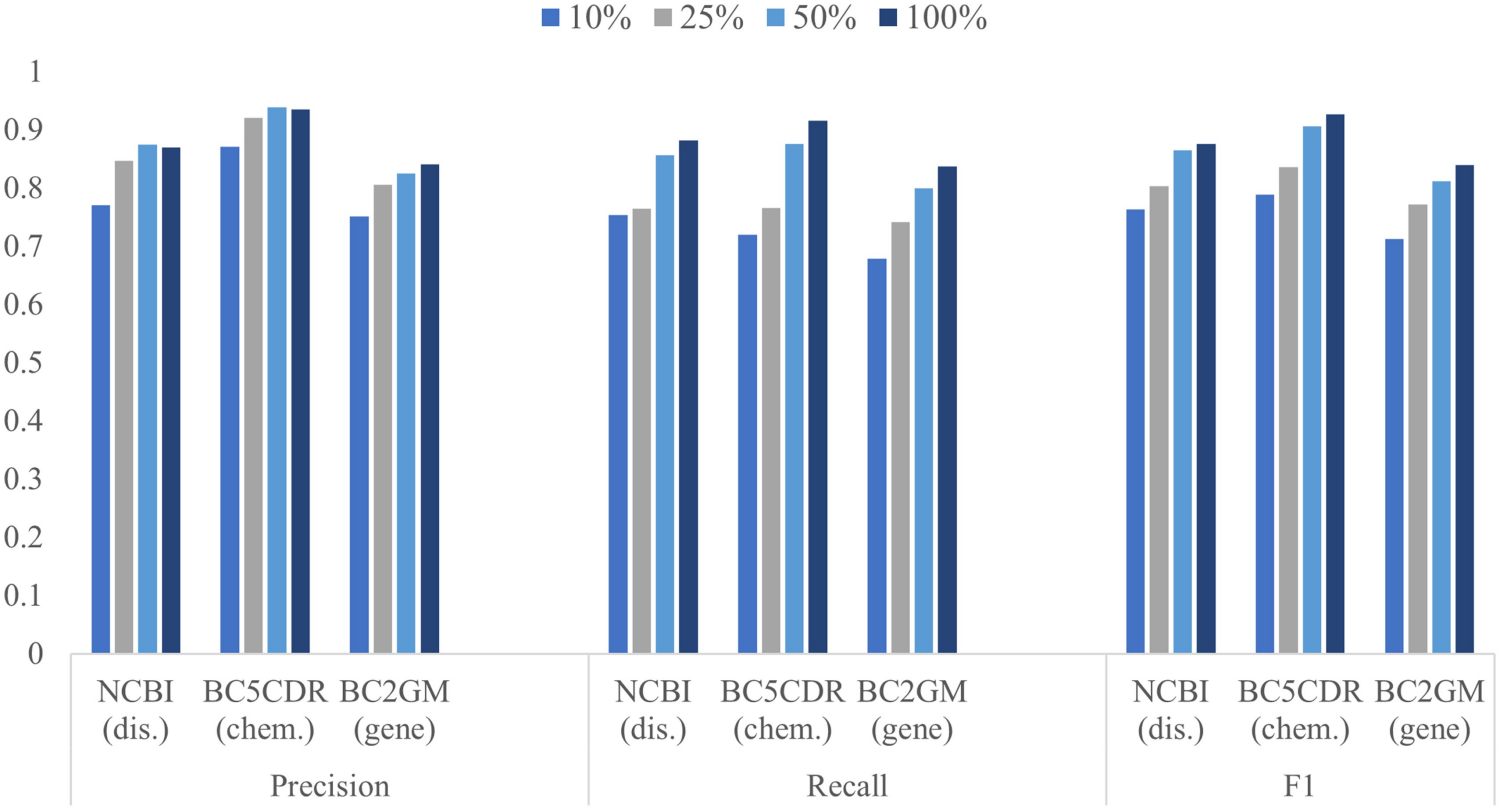
Precision, recall, and F1. Variations in values when the size of training dataset is reduced to 10%, 25%, and 50% compared to full training data for BioNER-LLaMA

Figure 4 shows the performance of the BioNER-LLaMA1 model when the size of the instruction dataset used for instruction tuning is reduced to half, quarter, and 10%. When reduced to half the dataset consisted of 11 242 instruction demonstrations with a total of 12 680 tagged mentions. The numbers for the 25% and 10% are 5618 instructions with 6268 mentions and 2248 instructions with 2497 mentions, respectively. When the size is reduced to half the drop in performance ranges from 1.1% to 2.7%. With 10% data, the performance drop is in the range of 11.3% to 13.8%. We note that the instruction demonstrations are randomly selected, a better selection strategy could improve the performance with the same amount of data or even a much lesser amount of data.

We also evaluated the effect of training data size on PubMedBERT models and compared it with BioNER-LLaMA. For the NCBI disease data, the performance dropped by 4.3% for 10% training data, 5.4% for 25% training data, and 3.5% for 50% training data compared to BioNER-LLaMA. For the BC2GM dataset, the drop in performance of the finetuned PubMedBERT models was in the range of 1.6% to 3.1%. As observed with the full training data, for BC5CDR-Chemical dataset, PubMedBERT models continued to perform better than BioNER-LLaMA for reduced size of training data. While 50% reduction resulted in only 0.4% difference, for 10% the difference was 8.5%.

#### 4.3.2 Single dataset versus multiple dataset instruction tuning

The model tuned using only the NCBI disease data (Table 3) achieved an F1-score of 0.762, whereas BioNER-LLaMA1 attained an F1 of 0.876 when evaluated on the same dataset. Generally, instruction tuning with multiple data sources produced higher performance, with the exception being BC2GM showing no difference versus its single-dataset counterpart. The BC2GM dataset contains roughly three times as many entities as the second largest dataset (BC5CDR-Chemical), suggesting its large size may account for the observed result wherein unified tuning did not further boost scores.

**Table 3.** Performance of single dataset instruction tuned model compared to BioNER-LLaMA.

Dataset	BioNER-LLaMA (P/R/F1)	Single dataset instruction tuned model (P/R/F1)
NCBI (disease)	0.869/0.882/0.876	0.869/0.678/0.762
BC5CDR (chemical)	0.935/0.916/0.926	0.926/0.847/0.885
BC2GM (gene)	0.841/0.837/0.839	0.838/0.837/0.837

#### 4.3.3 Effect of enhanced prompt on performance

With additional information added to the prompt describing in detail the entity to be extracted, there was no noticeable effect on the performance of the instruction tuned model (Table 4). While task-specific enhanced prompts have demonstrated improvement in model performance on zero-/few-shot approaches, our experiments with instruction tuning did not show a similar trend.

**Table 4.** Performance comparison of instruction tuned models with generic prompt versus enhanced prompt.

Dataset	Generic prompt	Enhanced prompt
NCBI (disease)	0.869/0.882/0.876	0.867/0.877/0.872
BC5CDR (chemical)	0.935/0.916/0.926	0.942/0.909/0.925
BC2GM (gene)	0.841/0.837/0.839	0.839/0.828/0.833

## 5 Discussion

In this study, we evaluated an instruction tuning framework that leverages existing biomedical NER datasets to automatically construct instruction datasets. BioNER-LLaMA demonstrated for the first time that a general domain LLM, instruction tuned on NER datasets can achieve performance on par with a carefully fine-tuned PubMedBERT model and a medical-specific LLM, PMC-LLaMA, both of which are pretrained on PubMed abstracts. One advantage of the instruction tuning framework is the relative ease with which existing datasets can be converted into instruction samples and combined to create new instruction datasets. By modifying the prompts according to the task and domain we can easily utilize the model for other information extraction tasks such as relation extraction and temporal event extraction which is not possible with domain specific finetuned BERT models.

While LLMs have some advantages there remain some limitations that need to be noted. First, developing and deploying LLMs can be challenging as they require significant computational resources to perform training, fine-tuning, and inference. High energy consumption and a large carbon footprint are other concerns. With parameters ranging into billions, LLMs can also pose storage capacity challenges. To fine-tune the LLaMA 7B model on the 22K dataset to generate BioNER-LLaMA, it required approximately 33 minutes using four 80 GB A100 GPUs. PMC-LLaMA is a 13B model and the knowledge injection training process involved utilizing 32 A100 GPUs. On the other hand, PubMedBERT is a 110M parameter model, which can run on a single GPU or even a CPU for smaller batch sizes making it a practical choice for environments with limited resources. Even though a general domain LLM, instruction tuned on NER datasets achieved performance on par with PubMedBERT, results from the LLMs did not outperform PubMedBERT results by a huge margin and hence a paired permutation test didn’t show statistical significance.

The lower performance of the GPT models (Table 1) can be attributed to several facts including (i) using basic prompts rather than task-specific enhanced prompts and (ii) selection of random samples for few-shot with the worst possibility that those samples may not include any entities. Our experiments on clinical NER datasets (Hu *et al.* 2024) have shown performance increase when prompts are enhanced with task-specific information such as entity definitions and annotation guidelines. Even then the GPT models were not able to match the performance of fine-tuned BERT models. Another factor that might boost the performance is the number of few-shot samples. Currently, we have experimented with only five samples and maybe more samples might improve performance, but it is not always guaranteed (Chen *et al.* 2023). A recent work (Margatina *et al.* 2023) has explored this issue of identifying the most informative demonstrations for few-shot learning by formulating it as an active learning problem. Their experiments show that “similarity” algorithm that retrieves examples from unlabeled data pool that are semantically similar to a test query sample achieved better performance than random and other sampling techniques. Additionally, running several prompt variations on the three datasets for two models (twice each—zero and few shot) incurs a lot of additional cost to use the GPT models.

From Table 2 demonstrating the generalizability of the models on other NER datasets, we observe that for the BC5CDR disease dataset there is more than 10% difference between strict and partial F1 scores. We further investigated this difference by analyzing the model predictions and the annotation guidelines (https://biocreative.bioinformatics.udel.edu/media/store/files/2015/bc5_CDR_data_guidelines.pdf) for the BC5CDR dataset. We found that there are two rules in the annotation guidelines, the fine distinction of which is missed by the model, resulting in predictions that do not match the gold annotations. These rules are (i) annotate the most specific disease mention (e.g. *partial seizures* preferred over *seizures*) and (ii) annotate minimum necessary text span (e.g. select *hypertension* instead of *sustained hypertension*). Performing error analysis, we found that the model always predicts the longest phrase corresponding to the disease mention (e.g. transient hyperammonemic encephalopathy, decompensated liver disease) resulting in boundary issues when compared to gold annotations. Some other rules in the annotation guidelines such as “DO NOT annotate general terms such as disease, syndrome, deficiency, complications, etc. However, terms such as pain, cancer, tumor, and death should be retained. DO NOT annotate references to biological processes such as tumorigenesis *…*” have resulted in other errors (missing and wrong predictions).

Like the BC5CDR disease dataset, the performance of all models on the NLM Chem dataset was also subpar. In this case, boundary issues were minimal, instead the majority of the errors resulted from abbreviations of chemicals. Both the models failed to predict almost all mentions of chemical formulas of elements (e.g. P, K, N) and the dataset contained a lot of these mentions. For example, N (nitrogen) was predicted not to be a chemical 31 times, O (oxygen) 36 times, and S (sulfur) 55 times. The annotation guidelines for NLM Chem specify annotating chemical/biochemical families and classes such as all concepts listed as descendants of organic and inorganic chemicals, polymers, carbohydrates, lipids, amino acids, etc. All mentions of amino acid(s) are annotated in the dataset along with different amino acids like lysine (abbreviated as Lys) and glutamic acid. Both the models predicted all amino acids as “O” indicating not an entity. Apart from that, fish-oil (38 mentions) was predicted as chemical entity by the PubMedBERT model while BioNER-LLaMA rightly predicted it as not an entity. In short, the lower performance can be attributed to challenges with abbreviation disambiguation which still remains a challenging problem in biomedical domain.

Our future work aims to further improve the efficiency of the proposed framework. Recently, several techniques were proposed to decrease the computational and storage costs. Low-Rank Adaptation (LoRA) (Hu *et al.* 2021) while keeping the pre-trained weights frozen, injects trainable smaller low rank matrices thereby reducing hardware barrier, storage requirement and task-switching overhead significantly. Parameter-Efficient Fine-Tuning (PEFT) (https://github.com/huggingface/peft) techniques only fine-tune a small number of (extra) model parameters, greatly reducing the number of trainable parameters for downstream tasks. Zhang *et al.* (2023) proposed LLaMA-Adapter with only 1.2M learnable parameters along with a LLaMA 7B model with frozen weights reducing the fine-tuning time to less than an hour on 52K instruction demonstrations. With quantization methods (Dettmers *et al.* 2022) showing reduction in memory footprint of LLMs, Dettmers *et al.* (2023) demonstrated the possibility of fine-tuning a quantized 4-bit model without performance degradation on a single GPU. Their method (QLoRA) (Dettmers *et al.* 2023) fine-tuned a LLaMA 65B model with GPU memory less than 48 GB compared to an estimated over 780 GB when using regular 16-bit fine-tuning. In future, we plan to develop a single model that can generalize and perform competitively on a wide range of biomedical NLP tasks. The above-mentioned techniques will be highly relevant in achieving this with the limited computational resources at our disposal.

We also plan to integrate self-instruct-based approaches for the entities with minimal annotations. In this work, we transform high-quality annotations from the existing benchmarks into instructions. However, in cases where entities only have minimal annotations, we could further integrate self-instruct-based approaches using LLMs into our approach. Starting from a small seed of human-generated instruction demonstrations, LLMs can be used to generate additional samples for training. It is challenging to ensure the quality and reliability of these generated instruction samples, as the performance of the models will heavily rely on the accuracy of these samples. Special care must be taken in curating and validating the generated instructions to maintain high standards of quality. We consider it as our future work.

## 6 Conclusion

In this study, we proposed and evaluated an instruction tuning paradigm that transforms biomedical NER from a sequence labeling task into a generation task and developed BioNER-LLaMA using LLaMA 7B as the backbone. The results show that—for the first time—a general domain LLM achieved comparable performance as that of fine-tuned domain-specific biomedical NER models and better than LLMs developed specifically for medical applications. It further shows that the proposed approach has great potential to transform the development and paradigm of biomedical NLP tasks.
